# Epulis

**Published:** 1872-06

**Authors:** 


### ARTICLE IX.

### *Epulis.*

The patient was a boy seven years of age, who had an epulis removed from his lower jaw about six months before. At the time of the operation, which was performed by Prof. Gross at the clinic, the bone was denuded of gum at the seat of the disease, and carefully scraped. A perfect cure has resulted. He now exhibits a similar growth on the opposite side of the mouth, from the gum of the upper jaw at the site of the second molar tooth; which was drawn two weeks before on account of the pain. The tumor is rather firm in consistence of a higher color than the surrounding gum, and is very tender and vascular, bleeding to the touch.

This variety of tumor (epulis) is probably of a sarcomatous nature, in some cases malignant, rapidly assuming the characteristics of epithelial cancer; in others benign, not returning after careful extirpation. The epulis does not originate in the gum, but in a socket of a tooth, generally one of the molars, from which it grows, causing the tooth to loosen and fall out. It need not attain a great size in order to seriously interfere with mastication and speech. If left alone it finally ulcerates; has a constant discharge; the neighboring lymphatics become involved, and the system becomes contaminated, and finally succumbs. It is useless merely to pare away this tumor from the gum, as it would inevitably return. The disease is seated in the osseous structure, and the only way to relieve it is to remove the affected part of the bone, either by scraping as in caries, or by sawing out a piece of the jaw. This treatment has been found in many cases to be the only one that gave the desired result.

After administering the anæsthetic, the tumor and gum, at the affected part, were removed with a chisel. The bone was exposed and well scraped. The disease appeared to be circumscribed. The permanent tooth was seen at the bottom of the alveolus. To check the bleeding, which was considerable, Monsel's solution was used; and a piece of raw cotton wet with it, pressed into and allowed to remain for some time in the wound. Prof. Gross considers this solution of the subsulphate of iron to be the best hæmostatic. But it must be used with care in operations on the mouth or tonsils, as it forms large coagula, which might be drawn into the larynx and suffocate the patient. This accident happened to no less a man than Bilroth, whose patient at his clinic, drew into his larynx a large clot of blood. The surgeon immediately performed tracheotomy, but the patient nevertheless died.—*Med. and Surg. Reporter.*

